# Building health workforce capacity for planning and monitoring through the Strengthening Technical Assistance for routine immunization training (START) approach in Uganda

**DOI:** 10.1016/j.vaccine.2019.04.015

**Published:** 2019-05-09

**Authors:** Kirsten Ward, Steven Stewart, Melissa Wardle, Samir V. Sodha, Patricia Tanifum, Nicholas Ayebazibwe, Robert Mayanja, Henry Luzze, Daniel C. Ehlman, Laura Conklin, Molly Abbruzzese, Hardeep S. Sandhu

**Affiliations:** a Centers for Disease Control and Prevention, 1600 Clifton Rd NE, Atlanta, Georgia 30329, United States; b Formally, Centers for Disease Control and Prevention, 1600 Clifton Rd NE, Atlanta, Georgia 30329, United States; c African Field Epidemiology Network (AFENET) Secretariat, Wings B & C, Ground Floor, Lugogo House, Plot 42 Lugogo House, Lugogo Bypass, Kampala, Uganda; d Formally with the Uganda National Expanded Program on Immunization, Ministry of Health, Uganda; e Uganda National Expanded Program on Immunization, Ministry of Health, Uganda; f The Bill & Melinda Gates Foundation, 500 Fifth Ave North, Seattle, WA 98109, United States

**Keywords:** Vaccination, Immunization programs, Health workforce capacity building, In-service training, Mentoring, Program evaluation

## Abstract

**Introduction:**

The Global Vaccine Action Plan identifies workforce capacity building as a key strategy to achieve strong immunization programs. The Strengthening Technical Assistance for Routine Immunization Training (START) approach aimed to utilize practical training methods to build capacity of district and health center staff to implement routine immunization (RI) planning and monitoring activities, as well as build supportive supervision skills of district staff.

**Methods:**

First implemented in Uganda, the START approach was executed by trained external consultants who used existing tools, resources, and experiences to mentor district-level counterparts and, with them, conducted on-the-job training and mentorship of health center staff over several site visits. Implementation was routinely monitored using daily activity reports, pre and post surveys of resources and systems at districts and health centers and interviews with START consultants.

**Results:**

From July 2013 through December 2014 three START teams of four consultants per team, worked 6 months each across 50 districts in Uganda including the five divisions of Kampala district (45% of all districts). They conducted on-the-job training in 444 selected under-performing health centers, with a median of two visits to each (range 1–7, IQR: 1–3). More than half of these visits were conducted in collaboration with the district immunization officer, providing the opportunity for mentorship of district immunization officers. Changes in staff motivation and awareness of challenges; availability and completion of RI planning and monitoring tools and systems were observed. However, the START consultants felt that potential durability of these changes may be limited by contextual factors, including external accountability, availability of resources, and individual staff attitude.

**Conclusions:**

Mentoring and on-the-job training offer promising alternatives to traditional classroom training and audit-focused supervision for building health workforce capacity. Further evidence regarding comparative effectiveness of these strategies and durability of observed positive change is needed.

## Highlights

• Enhanced supportive supervision used to improve immunization planning and monitoring.
• On-the-job training and mentoring improved health care worker motivation.
• Routine immunization systems were strengthened by this approach.
• In-service training is promising for building immunization workforce capacity.

## 1 Introduction

Vaccination is among the most cost-effective public health interventions, due to low cost of traditional vaccines and potential to save lives [1], [2]. Globally, coverage for the third dose of diphtheria-tetanus-pertussis (DTP) vaccine (DTP3) is the standard indicator of performance for utilization of the routine immunization (RI) system [3]. From 2010 to 2016 DTP3 coverage plateaued at 84%–86% globally, equating to 19.5 million under- or un-vaccinated children in 2016, mostly from 10 low- and middle-income countries (LMICs) [4]. A major contributing factor to this is routine immunization (RI) systems that do not function as intended [5], [6].

Strengthening RI systems is one of the goals of the Global Vaccine Action Plan (GVAP), with improving sub-national planning, monitoring, and workforce capacity as a key components [7], [8], [9], [10]. Such efforts are focused in LMICs, which have a high number of unimmunized children and disproportionately poorer health systems [11]. Health care workers (HCWs) are increasingly expected to have general and specialized technical competencies to meet the growing complexity of RI service delivery and integration with other health interventions [9], [12], [13]. Pre-service education and training of the health workforce focuses on building specialized knowledge and skills [14]. In-service training of health professionals already employed aims to maintain technical knowledge and skills, develop those required to implement processes specific to the position and keep pace with continuing changes in policy and practice [14], [15].

Approaches to strengthen HCWs’ competency through in-service capacity building in LMICs have taken different forms, the most common being traditional, didactic, group, or cascade-style training held outside the workplace [12]. However, available evidence highlights limitations of these approaches such as removing HCWs from their work setting, targeting HCWs in management positions who do not perform the skills being taught, not addressing individual learning needs or, accounting for participants’ previous experiences, a potential to be costly, and incomplete attendance [9], [12], [16]. In addition information might change as it moves through cascade-style approaches. In-service training that utilizes adult learning principles [17], including on-the-job training, mentoring and feedback, and follow-up has been shown to increase job satisfaction and health worker motivation [12], [16], [18], [19]. Supportive supervision visits are an opportunity to implement such training approaches, although in LMICs, these visits have traditionally focused on auditing of resources and delivery of new information about multiple health topics in a short period of time with infrequent follow-up [16], [20]. The benefits of supportive supervision can be maximized by incorporating training, mentoring, and regular follow up; by focusing on the needs of individual HCW roles; and being delivered by staff with technical competence and strong interpersonal skills. In addition, further definition and evaluation of the effectiveness of supportive supervision approaches is necessary [12], [16].

The Strengthening Technical Assistance for Routine Immunization Training (START) approach aimed to utilize practical training methods to help build supportive supervision skills of district staff of the Uganda National Expanded Program on Immunization (UNEPI) and build skills of district and health center staff on key RI planning and monitoring activities. START was initially implemented in Uganda, one of 10 countries with the most un-immunized children in 2012 [21], through a collaboration between the UNEPI, U.S Centers for Disease Control and Prevention (CDC), the African Field Epidemiology Network (AFENET) and the Bill & Melinda Gates Foundation. Within the context of the RI system there is limited evidence about the use, or evaluation, of in-service workforce capacity building strategies used in the START approach, including mentoring and on-the-job training. To address this gap, this paper describes the design, activities, and program monitoring results from the initial implementation of the START approach during 2013 and 2014 in Uganda.

## 2 Methods

### 2.1 Design

The START approach to in-service training was designed to build individual capacity [22] of (a) district UNEPI staff to use practical training techniques in supportive supervision visits and, (b) both district EPI and health center staff to plan and monitor RI activities. There were three key implementation principles of the START approach: (1) work within the context of the existing health system utilizing experiences of external consultants to conduct focused training on key elements of RI planning and monitoring as prioritized by UNEPI; (2) conduct repeat visits to districts and health centers to reinforce learning and support on-going application of knowledge and skills; (3) provide a collaborative, friendly, and non-fault finding approach. Key topics of focus included RI micro-planning [23], using data to prioritize health centers to target for supportive supervision, improving quality of administrative vaccination data, RI performance monitoring (i.e., tracking uptake of vaccines), and tracking defaulters (i.e., children who had missed doses of vaccine). Logistical, managerial and operational aspects of the START approach are summarized, from the implementation experience, in Table 1.

**Table 1:** Key logistical, managerial and operational elements of the Strengthening Technical Assistance for Routine Immunization (START) approach in Uganda, July 2013 – December 2014a.

*Logistical and managerial*
• Availability of dedicated vehicles with reliable driver for the START consultants to conduct their work • Funding for START consultants’ stipend, fuel, and district staff lunch allowance • Availability of UNEPI program planning and monitoring tools (e.g., tally sheets, monthly HMIS reports), rapid data quality assessment tool, brief lesson plans on each major topic for use during health center visits^b^ • Availability of in-country supervisor/mentor for START consultants

*Operational*
• Training for START consultants pre-deployment • Provision of explicit guidance to START consultants on training methodologies to use during health center visits^b^ • Allocating a geographical area for START consultants to work, including selection of 5–6 low performing (i.e. low immunization coverage) health centers per district in a maximum of 4-5districts ^**^ • Meeting with national stakeholders and district leadership prior to each 6 month deployment to introduce the START approach and START consultants, obtain buy-in from district leadership, and clarify roles and responsibilities^b^. • Meeting with at the end of, each 6 month deployment to share experiences with implementation, outcomes of the work and identify areas for improvement^b^ • On-the-job training and mentorship for district UNEPI staff (i.e. immunization focal person) by START consultant including jointly conducting supportive supervision visits to health centers that included on-the-job training and mentorship of health center staff • Repeat visits (i.e. more than one visit) to all selected districts and health centers • Working with a district counterpart who spoke the local dialect • Use of data (i.e. immunization coverage) from the district and health centers’ to demonstrate planning and performance monitoring skills and processes • Developing focused terms of reference for the START consultants work • Implementation of routine monitoring activities^b^ focused on process indicators and short term outcomes to inform - Provision of templates and training for START consultants to record and report monitoring data - Routine reporting and validation of reported START consultants activities - Observational visits by supporting partners at least once per START team - Organizational assessment^c^ of RI system resources^b^

a Developed from collation of feedback from the START consultant interviews, field observations and internal discussions with staff managing the START approach in Uganda.

b These elements were amended as a result of feedback from the START consultant interviews and field observation of their work. The optimal approach is listed.

c Organizational assessment was a semi-structured questionnaire which aimed to measure presence of RI planning and monitoring tools and extent of implementation of RI systems in all districts where START consultants worked and, within these, a selected number of health centers.

**Fig. 1:**
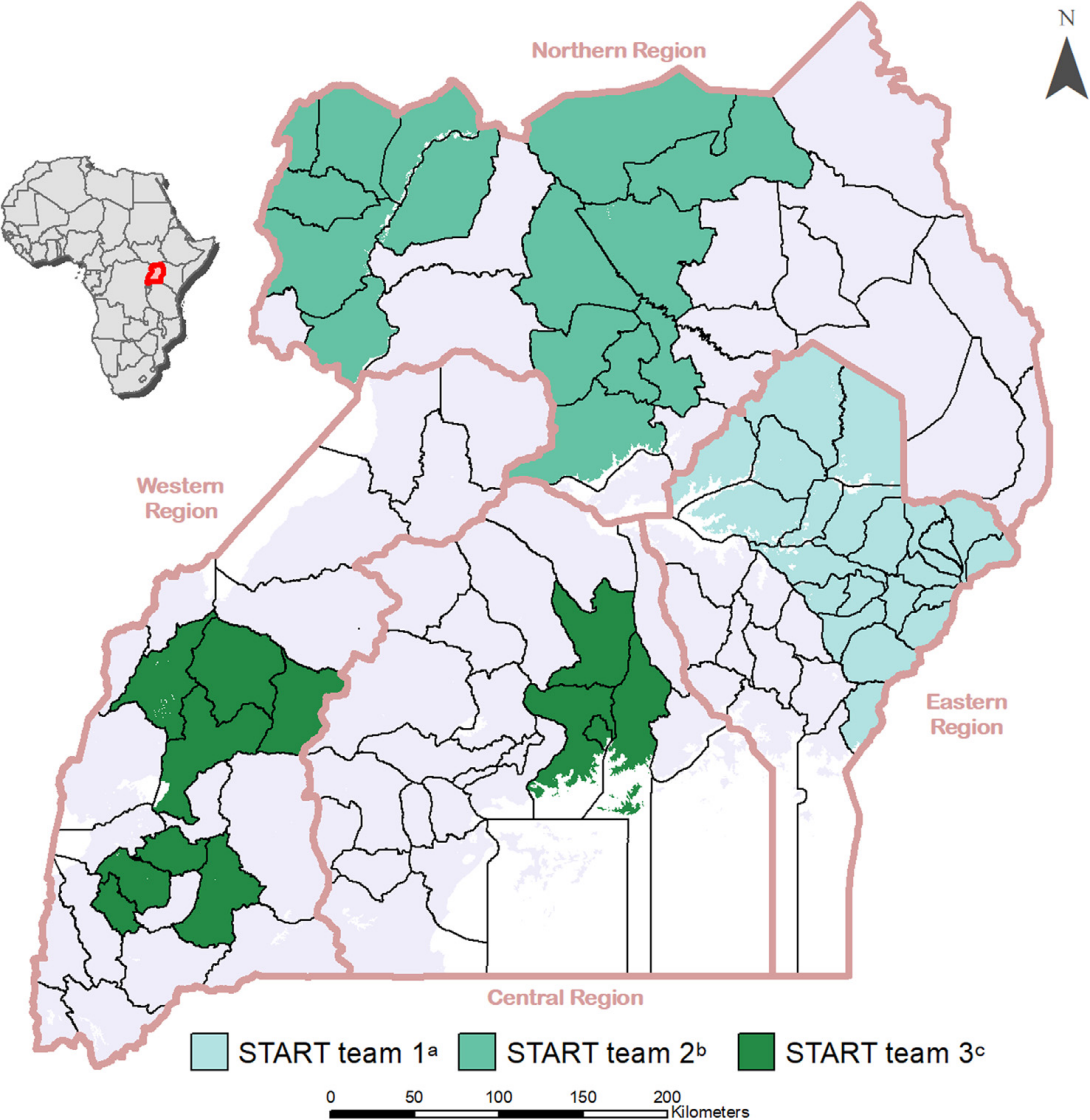
Geographic location of the districts covered by the three Strengthening Technical Assistance for Routine Immunization Training (START) teams in Uganda, July 2013 to December 2014. (a). START team 1: Four international consultants covered 22 Districts (20%) and 273 health centers from July to December 2013. (b). START team 2: Four international consultants covered 16 Districts (14%) and 160 health centers from February to June 2014. (c). START team 3: Four international consultants covered 11 Districts and the five Divisions of Kampala district (10%), and 359 health centers from July to December 2014.

Three different START teams consisting of four international consultants (START consultants) were deployed in Uganda from July 2013-December 2014 (Fig. 1). The criteria for selection of START consultants included both technical and soft skills and aligned with the guiding principles and topics of focus for START. The initial selection criteria for START consultants included, prior experience in planning and monitoring immunization program activities at sub-national or national level; delivering on-the-job and in-service training on immunization to a variety of pubic health professionals; evidence of use of a collaborative and friendly approach to training and supervision; and working outside their home country. The criteria for selection of START consultants evolved over the course of recruiting several START teams; it became clearer that familiarity with a multiple and varied RI topics, effective on-the-job training skills and enthusiasm for training were key attributes for consultants to bring to START. Prior to deployment, all START consultants completed 7 days of pre-service training on technical elements of the RI system, techniques of mentoring and on-the-job training, and orientation to the UNEPI.

START teams were deployed in all four geographical regions of Uganda (Fig. 1). Each START consultant was assigned three to five low performing districts where they worked primarily with the district UNEPI staff. Selection criteria for low performing districts included low (<80%) DTP3 coverage, high (>10%) drop-out rates between the first and third dose of DTP vaccine, and/or outbreaks of measles in the district within the past two years. START consultants were provided rented vehicles, drivers and fuel to facilitate regular mobility.

Before the START teams were deployed, a meeting was held with leadership from the selected districts, including Health Directors, UNEPI staff, and START partner organizations to introduce the START consultants, gain support for the START approach, and develop a district-specific implementation plan, including selection of low-performing health centers, timeline for initial and re-visits to each. Before the end of the 6-month deployment, each START consultant met again with district leadership to plan for continuation of the START approach, to provide feedback on experiences and recommendations for strengthening ongoing planning and monitoring of the RI system. A similar meeting was also held with each START team, UNEPI staff and other national stakeholders, where results of routine monitoring (reported below) were shared and discussed.

From the outset, START consultants worked with district UNEPI staff to identify 5–6 low-performing health centers in each district to visit. The criteria for selection of low-performing health centers was the same as that used for the districts. In selected health centers, START consultants worked jointly with district UNEPI staff, and used an approach that included supportive supervision and on the job training to build HCW skills and to foster health facility processes to strengthen key elements of planning and monitoring for the RI system that could continue to function after training from the START consultant was finished (START approach). Tools used to facilitate the START approach included the UNEPI template for RI microplanning and standard administrative vaccination data collection forms (i.e, monthly reports of vaccines submitted to the health management system, tally sheets for vaccination sessions, and health facility immunization register). A new questionnaire was developed to assess the availability and congruence between different sources of administrative vaccination data reported in the immunization information system. The START approach was designed to (1) minimize the HCW’s time away from their regular duties; (2) involve all interested facility staff to enhance continuity and sustainability; (3) tailor content to address individual HCW and facility organizational gaps; (4) allow for use of interactive training methods (e.g., discussion, demonstration, and hands-on practice); (5) provide an opportunity for repeat exposure to knowledge and skills and to practice new skills in the context in which they would be eventually be performed.

During the initial visit to health centers, START consultants modeled the START approach to district UNEPI staff; during follow-up visits, they mentored and encouraged district UNEPI staff to lead the START approach at health centers to enhance their retention of knowledge and skills. START consultants and district UNEPI staff conducted at least one follow-up visit to all selected health centers in the districts where they worked. More frequent follow up visits were conducted if needed (i.e. change in staff, further support to address gaps in learning). The follow-up visits provided the opportunity to cover topics in more detail at different visits, re-inforce previous learning, address gaps in learning and motivate staff, all of which increases the likelihood of retention and implementation of the learning [18].

When requested by district leadership, START consultants supported other RI activities (e.g., national mass immunization campaigns). In addition, when requested by district leadership, START consultants occasionally led or presented at one time off-site group training workshops in order to provide the opportunity for staff from health centers not targeted for the START approach to receive training on RI planning and monitoring.

### 2.2 Monitoring of the START approach

Implementation of START in Uganda was monitored using a mixed methods utilization-focused methodology [24], [25], [26], incorporating elements of improvement science [27] which evolved with each team. As this was a new initiative, monitoring focused on the process and outputs with the aims of improving the approach and understanding short-term achievements.

Each week, START consultants reported location, type, and frequency of their activities to CDC through a standardized spreadsheet with the names of districts and health centers where START activities were conducted, presence and role of national and district staff in these activities (START team 1 and 3 only), type of activity, and topics covered during each activity. Activity type was defined based on anticipated approaches to training, either group or on-the-job. Topics were defined based on the elements of RI planning and monitoring on which START activities were focused: microplanning, administrative data recording and reporting, using administrative data for action, vaccination monitoring chart, use of the Reaching Every District (RED) tool to prioritize health centers for supportive supervision [28], and defaulter tracking (START team 2 & 3 only). CDC staff collated and descriptively analyzed the START consultant activity data using SAS 9.3 [29], [30]. Results of the analysis were sent to START consultants for verification and data were amended to best reflect actual events.

In the latter half of each team’s deployment, CDC staff members accompanied each START consultant to observe their work, and conduct individual semi-structured interviews with them. Interview questions were predominantly open-ended and aimed to elicit START consultants’ feedback about implementation of the START approach, adaptations to improve the approach, and perceived effects of their work.

To further understand change in RI planning and monitoring tools and systems, resulting from implementation of the START approach, semi-structured organizational-level assessment (OA) questionnaire was implemented at all districts and the selected health centers visited by START Uganda team 3. The OA aimed to measure presence of RI planning and monitoring tools and extent of implementation of RI systems using both closed and open questions. OAs were conducted for all districts where START consultants worked and in a minimum of five purposively selected health centers in each district; at least one health center from each of the four types of health centers in the Ugandan health service classification system [31]. OA’s were conducted at the START consultants first visit (before implementation of the START approach) and their last visit (after implementation of the START approach) to each district and selected health centers. OAs were completed in hardcopy, entered into an online version of the OA from which CDC staff downloaded and descriptively analyzed data using SAS 9.3.

CDC and the UNEPI considered the monitoring activities for START approach to be routine public health program evaluation thus full institutional review board approval was not required.

## 3 Results

Results from the three START teams deployed in Uganda during July–December 2013 (Team 1), January–June 2014 (Team 2), and July–December 2014 (Team 3) (Fig. 1) were aggregated to document outputs from the START approach in Uganda, except when differences between teams were used to highlight key lessons learned.

The three START teams reached 50 (45%) of the 112 districts in Uganda, including the five divisions within Kampala district. START team 1 consultants found that there was insufficient time to fully implement multiple on-the-job visits needed for the START approach in all selected health centers in the 22 districts. Thus, fewer districts were selected for START team 2 (n = 16) and START team 3 (n = 12). All 50 districts received at least one visit from the START consultant (median of six additional visits per district (Inter-quartile range (IQR): 5–9)) with the majority of districts (n = 50, 89%) receiving three or more visits. START consultants trained and mentored 162 district UNEPI staff (mean = 3 per district).

Together, START consultants and district UNEPI staff reached > 2000 HCWs in 792 health centers (median = 9 nine health centers per district); 410 (52%) of these were initially reached through on-the-job training visits (median = 7 centers per district) and 382 (48%) were initially reached through group training workshops (median = 13 centers per district). Health centers initially reached through group training received fewer repeat visits (9%, 34/382) compared with health centers initially reached through on-the-job training (58%, 238/410). Among all 792 health centers visited 444 (56%) received at least one on-the-job training visit (median: 2, IQR: 1–3), and among these, 59% (260/444) received two or more on-the-job training visits. The START consultants reported that visit frequency was influenced by geographical location of the targeted health centers, engagement of the district EPI staff, rate and extent of improvement in skills, and frequency of group training, which left less time for repeat visits.

**Fig. 2:**
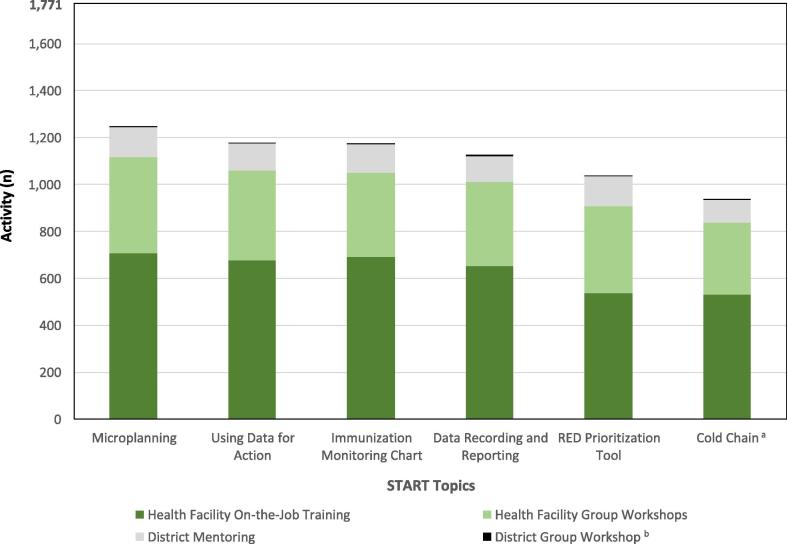
Frequency of implementation of training topics, by training method and health system level, delivered by three Strengthening Technical Assistance for Routine Immunization Training (START) teams in Uganda, July 2013 – December 2014. (a). Includes forecasting of vaccines and injection materials, recording wastage and usage in the stock book, and completing the temperature monitoring chart for the vaccine refridgerator. (b). A total of 21 District group workshops occurred across all START topics (range 3–4 per topic). District group workshops accounted for a total of 1.2% (21/1771) of all activities, the least of any type of training at district or health center level.

Across all three START teams 1771 activities were reported by START consultants. Activities that were not a START training activity or focused on a START specific topic were excluded (48 administrative activities and 86 “other” activities, including 35 activities that only supported SIA implementation). Of 1771 START training activities (group or on-the-job) reported, 366 (21%) were at the district-level and 1405 (79%) were at health center level. Of the district-level activities, 99% (361/366) were on-the-job mentoring and training, and the remainder (1%, 5/366) were group workshops for district staff. Two-thirds (67%, 939/1405) of health center activities were on-the-job training, and the remainder (33%, 466/1405) were group workshops for staff from different health centers (Fig. 2).

Across all reported START training activities (n = 1771), the most frequently covered RI planning and monitoring topic was microplanning (70%, 1247/1771), followed by those topics covering collection, monitoring, and use of administrative vaccination data (Fig. 2). Only START teams 2 and 3 reported covering the topic of defaulter tracing, which was covered in 800 (69%, 800/1159) activities reported by START teams 2 and 3. Nine START training activities (0.5%, 9/1771) reported covering the topic of SIAs in addition to START topics. START consultants reported that district and health center staff took longer to build skills in data-related topics, largely due to their technical nature. However, demand for these skills was high, given ongoing accountability for these data through the monthly Health Management Information System (HMIS) reporting process. Similarly, demand for knowledge and skills for planning and monitoring of vaccine and injection materials (e.g. forecasting, recording usage and wastage) was higher than anticipated. This topic was not included in the original list of key RI planning and monitoring topics identified by UNEPI.

In START teams 1 and 3, district UNEPI staff accompanied the START consultant to more than half of the health center training activities (54%, 493/920) (information not available for START team 2). START consultants reported that the capacity of the district UNEPI staff to accompany the START consultant was affected by the extent of competition for their time and district leadership’s support of the strategy. Additionally, some district EPI staff visited different health centers from the START consultants to increase the reach of the strategy and disseminate updated information about the EPI program, as was the usual focus for supportive supervision visits. District UNEPI staff took a lead role in on-the-job training at 30% of jointly conducted health center visits, though an upward trend was observed throughout the 6-month START deployment from 24% in the first month to 43% in the sixth (final) month.

Feedback from START consultant interviews and observational visits identified factors affecting implementation (Table 2). START consultants indicated that addressing the following factors was outside their control, though had the potential to enhance the START approach, competing priorities for district staff time; human, financial, and material resources allocated to EPI at the district; and external accountability from higher level management. During observational visits, district and health center staff reported that they valued repeated visits from the START consultants who helped clarify elusive concepts, brought meaning to RI activities, and generated internal motivation to develop and implement targeted skills. This finding is illustrated by the following comment:

“*This is the third time (since I began working that) we’ve been trained in (RI) micro-planning. The first time we were trained, it was not clear at all. The second time (the division) convened another training but still we never understood, and that is why we planned at the division without health centers first sharing their micro-plans. It is now with the START support that I have understood micro-planning and practiced it*.” (Nurse and EPI focal person, division of Kampala)

**Table 2:** Socio-ecological factors a affecting implementation of the Strengthening Technical Assistance for Routine Immunization Training (START) approach in Uganda, July 2013 – December 2014b.

*Social, political and Uganda National Expanded Programme on Immunization (UNEPI) environment*
• Predominant training styles used to train the health workforce - classroom-style group training • Culturally, staff performing the work do not often attend classroom-style group training for technical work, a privilege often reserved for those in leadership positions • Fluctuation in the number of operational districts and health centers and those who provide RI services, due to changing boundaries and funding • Availability of national-level health system strengthening funding for UNEPI • Occasional stock-out of vaccines which halted provision of routine immunization services • Community demand for immunization services was high, so staff were busy • UNEPI requirement that all districts complete annual microplans for routine immunization – affected demand for support to do this • UNEPI tools for planning and monitoring not available, incorrect, or not viewed as user-friendly • Frequent mass immunization campaigns, for which planning and monitoring is conducted have built workforce capacity for planning and monitoring, but reduced time for planning and monitoring, and supportive supervision, about RI activities) • Introduction of new vaccines into the UNEPI program (seen as opportunity to enhance planning and establish program monitoring, both of which START approach could support) • Hard-to-reach districts and health centers, due to geographical isolation, non-Government ownership or insecurity limited scope of START consultants work • Limited/no availability of, inaccurate, and competing sources of target population data which reduced utility of planning and routine monitoring

*Community*
• Differing relationships with and between: higher levels of health system, political leaders, community, staff, supervisors, non-government organizations in the health sector, other non-health sectors of government. Good relationships were critical for the START consultants work. • Anti-vaccination groups in the community affect demand for vaccination services and individuals workload in trying to overcome this challenge • Acceptance of a foreigner, both at work and in the broader community

*Organizational*
• Insufficient ownership and commitment to EPI at the district and health center level, resulting in poor allocation of resources to these activities • Competing priorities for funding and human resources, due to limited supply of both • High staff turnover at district and health center level • No means of transport for district staff to conduct supportive supervision, or competition for available transport • Requirement for additional allowance for movement outside of usual place of work • Management structure which did not afford training opportunities to staff not in management positions

*Interpersonal*
• Competing priorities for health care worker time • Poor relationships/perceptions of supervisors and/or co-workers

*Individual*
• Attrition of participants from classroom-style training, often in response to demands of their regular work • Perception of the value of the knowledge and skilling being taught • Lack of awareness of the need for, importance of, or barriers to planning and monitoring of UNEPI program activities • Low levels of knowledge and skills in UNEPI planning and monitoring • Many staff needed repeated exposure to, and application of, knowledge and skills • Insufficient salary which reduced staff motivation • External motivation main driver of action, though this was often limited by infrequent or inadequate supervision, infrequent requirement for planning data, and insufficient oversight of accuracy of administrative vaccination data received from health center or district level

a Framework used for reporting adapted from the Social Ecological Model [49].

b Developed from collation of feedback from the START consultant interviews, field observations and internal discussions with staff managing the START approach in Uganda.

Three months into their work, and after several re-visits, the most commonly reported changes observed by START consultants were related to positive staff motivation towards RI, completion of planning and monitoring tools, implementation of new systems for archiving, and checking of accuracy of vaccine administration data. START consultants felt their support had increased district and health centers’ awareness of the underlying reasons for challenges experienced, and what they could do to address or work within them.

START team 3 completed OAs in 16 districts (100%) and 90 health centers (17%). OAs were immediately used to inform the focus of the START consultant’s work with that district or health center. Improvement was seen in all indicators (Table 3), although the magnitude of change varied widely across districts (6%–88%) and health centers (20%–91%) . Availability and completion of planning tools (e.g., RI micro-plan) had a higher magnitude of change, as compared with monitoring tools (e.g. vaccination monitoring chart). Frequency of supervisory visits from district to health center, and provision of written feedback after these visits had higher baseline levels than other indicators, but showed little change over time.

**Table 3:** Change in routine immunization (RI) planning and monitoring tools and systems in districts and health centers in Uganda, visited by START team 3 July to December 2014.

	Baseline	Post	Difference^a^
	n (%)	n (%)	% points
*District (n = 16)*^b^			
Uganda National Expanded Programme for Immunization (UNEPI) routine immunization microplan is available	3 (19)	11 (69)	50
List of health centers in the District is available	15 (94)	16 (100)	6
Up-to-date target population < 1 year of age written down and accessible	11 (69)	16 (100)	31
Vaccination monitoring chart available with administrative vaccination coverage with data from the last 3 months	2 (13)	16 (100)	88
Criteria used by district staff to prioritize supportive supervision visits to health centers			
Reaching Every District (RED)[50] tool for prioritization of health centers for supportive supervision	8 (50)	16 (100)	50
New staff at health center	3 (19)	6 (38)	19
Low reported vaccination coverage	5 (31)	10 (63)	32
Reported problems at health center	12 (75)	14 (88)	13
EPI data quality assessed during supervisory visits to health centers	5 (31)	13 (81)	50

*Health centers (n = 90)*			
UNEPI RI microplan is available	2 (2)	74 (82)	80
List of parishes and up-to-date RI target population for each parish is available	6 (7)	88 (98)	91
Up to date target population < 1 year of age is written down and accessible	49 (55)	88 (98)	43
Vaccine forecasting documentation is available	1 (1)	82 (91)	90
Monitoring chart available with administrative vaccination coverage data from the last 3 months	5 (6)	84 (93)	87
Number of children vaccinated with each vaccine reported on the monthly health management information system report is validated for accuracy and completeness before the report is sent to the district	18 (20)	85 (94)	74
Health center has a defaulter tracking system	7 (8)	79 (88)	80
Supervisor from district or sub-district visited the health center within the past 3 months	49 (54)	67 (74)	20
Among those who received a visit from the district or sub-district, health centers where written feedback provided at the supervisory visit	19 (39)^c^	63 (94)^c^	—

a Difference in proportion between baseline and post organizational assessments.

b Districts covered by START Uganda team three include 11 districts and the five divisions of Kampala district (total 16 operational districts).

c Denominator is those health centers who had a supportive supervision visit from a district or sub-district staff member in the past 3 months.

## 4 Discussion

To be effective, in-service training of the health workforce should focus on their specific training needs, be job-based and practical, and involve regular follow-up to ensure knowledge and skills are understood and continually practiced. The START approach was designed to achieve this, using techniques of on-the-job training and mentoring within the platform of supportive supervision. Positive changes in staff motivation, awareness of challenges, availability and completion of planning and monitoring tools were observed at district and health center levels. However, START consultants felt the potential for sustainability of these and newly introduced processes was limited by various contextual factors, including limited external accountability for supportive supervision activities; competition for staff time; availability of material, human, and financial resources; and individual staff motivation.

The START approach used on-the-job training because it could be implemented as part of an existing supportive supervision platform and had strong evidence of positive learning outcomes [12], [18], [32]. Although focused on in-service training, time-limited on-the-job training has also been shown to be a valuable addition to pre-service training [15], [33]. On-the-job training has been used previously in Uganda with some success [34], [35], [36], though these approaches were largely one-time initiatives, did not focus on RI, were not part of a routine supportive supervision platform, and were no longer being implemented at the time the START approach was being considered. Although on-the-job training predominated across all teams, there was some variation in training methodologies used within and between START teams, which reflected tailoring of the START approach to meet local needs and circumstances.

While mentorship has been less frequently used and evaluated outside of clinical practice, it was incorporated in the START approach at the district level to enhance potential for sustainability. Mentorship in the START approach was done through development of a collaborative working relationship between START consultants and district EPI officers, on-site observation, and the provision of targeted and individualized feedback, which together have been shown to improve knowledge, skills, and motivation in mentees [12]. However, the less than optimal frequency of collaborative visits for on-the-job training of health center staff limited the opportunity to conduct mentorship with district UNEPI staff.

The START approach differs from previously reported in-service workforce capacity building strategies to strengthen RI systems in the African region, which have favored several days of off-site classroom style training on a broad range of topics related to program management, vaccine management and delivery, vaccination program planning, and monitoring of RI [37], predominantly targeting national or regional level staff. In contrast, START consultants conducted on-the-job training solely at sub-national and service delivery levels, where they were embedded within the fabric of the district workforce for 6 months and focused on specific RI planning and monitoring topics. In this context, START consultants used existing tools, resources, and experience to build the capacity of district and health center staff and enhance some components of existing RI systems to meet program requirements for planning and monitoring, which was the focus of START approach in Uganda as requested by UNEPI. As a result of the focus on planning and monitoring, skills in implementing other areas of the RI system, such as procurement, management and delivery of vaccines and surveillance of adverse events following immunization were not addressed formally through on-the-job training or mentorship provided by the START consultants. Focusing on additional or different topic areas could be considered by countries implementing the START approach in the future.

Strengthening supportive supervision is a key strategy to build and sustain workforce capacity at individual and organizational levels [12], [19], [34], [36], [38]. Routine, external supportive supervision has been reported to help staff tackle complex problems and problems over which they have little control [36]. Benefits of routine supportive supervision have been observed in previous workforce capacity building initiatives in Uganda [34], [35] and other countries in the African region [9]. Regular review meetings of health center staff held at the district level are reported to provide similar benefits as supportive supervision but cannot be tailored to individual learning needs. However, if well-used, review meetings can provide a platform for training, and can promote peer-to-peer learning, information sharing and use of data, all of which strengthen accountability and motivation of staff [38]. In addition, these meetings could provide opportunity to inform national-level decision makers of priority topics for sub-national staff training, which would help inform the design of capacity building initiatives such as START. Despite evidence of success, the frequency and quality of interactions required for optimal supportive supervision remains unclear [12]. In addition, robust evidence of longer-term effects of supportive supervision is lacking because of the complexities in conducting controlled evaluations of multifaceted practical training initiatives implemented in real-world settings [9], [12], [16].

The financial, material, and human resource shortages reported to inhibit the ability of district UNEPI staff to implement routine supportive supervision visits are similar to those reported in other LMICs [16], [39], [40], [41]. START consultants brought additional resources (e.g., a vehicle) to temporarily remove some barriers to conducting supportive supervision visits. In addition, repeat visits, as a key component in the START approach, strengthened supportive supervision by providing accountability to adhere to protocols, re-enforce learning, and address knowledge gaps, all of which were reported to increase staff motivation and confidence to implement the capacities they had built. However, numerous human resource factors outside of the START consultants control (Table 2), including competing priories on staff time and staff turnover, led to less than optimal number of joint supervision visits by the district UNEPI staff and START consultant. Sustaining improvements achieved through the START approach will require strengthening of current systems for routine supportive supervision in Uganda. Elements to be strengthened include, routine involvement of sub-national staff in identification and prioritization of their learning needs, ongoing training of existing and new district UNEPI staff in practical supportive supervision techniques and routine and timely access to adequate material, financial and human resources to implement routine supportive supervision, regardless of the topic of focus. At the same time, understanding the array of complex contextual factors effecting routine supportive supervision and the broader RI systems, then developing solutions to address the root causes of these require more than technical solutions alone. Such efforts should be led and owned by the relevant decision-making bodies within the county (e.g. Government).

Since the implementation of START Uganda, the START approach has been implemented in three additional low- and middle income countries [42]. In each of these countries, country-nationals were hired as START consultants. This was done with a view to enhance the potential for sustainability of the strategy though building mentoring knowledge and skills of country staff and reducing cultural and language barriers. In addition, the length of START teams’ deployment was extended from the initial 6 months in Uganda to 8–9 months. This was done to allow additional time for on-the-job training and more repeat visits (where needed) to both districts and health facilities.

### 4.1 Limitations

Monitoring was built into the START approach from the beginning and collected self-reported data to inform adjustments to the START approach and measuring effects of the START consultants work in selected districts and health centers [43], [44]. There are limitations to individual methods used for this, though in combination, they were able to obtain the desired evidence. These self-reported data in activity monitoring and OAs are potentially influenced by responder bias, though these data were routinely reviewed by CDC staff to verify completeness and later verified with the START consultants to enhance accuracy. There was also potential for responder bias in consultant interviews, although these were conducted individually, with explanatory statements before each interview to emphasize that the purpose was to improve the START approach [45]. There was potential for the Hawthorne effect to positively skew field observational data [46]. Triangulation of observational data with other monitoring and evaluation data was done to identify possible observation bias, however, due to high congruence between these data sources observer bias appeared limited. Whilst improvement in vaccination coverage was not the main outcome of START approach, theory of change suggests that building workforce capacity as a component of RI system strengthening has the potential to improve administrative vaccination coverage. However, longer-term outcomes, such as administrative vaccination coverage, are influenced by a broader range of factors which could not be decisively measured, controlled for, or attributed solely to the START approach, a known challenge in program evaluation of health initiatives [44], [47], [48].

## 5 Conclusion

A stable, availabe, well-trained and motivated health workforce; sufficient financial and material resources; and routine implementation of supportive supervision and accountability measures would help to optimize RI planning and monitoring activities. Despite the GVAP recommendation to strengthen sub-national planning, monitoring, and workforce capacity as well as extensive investment in in-service training of health care workers using class-room style didactic approaches, further evidence of optimal methods for, and the longer term effects of, in-service workforce capacity building is needed. Current evidence suggests a move away from group, didactic, classroom-style training of health care workers and audit-focused supportive supervision in LMICs would enhance outcomes and sustainability of these interventions [12], [16]. As immunization services are integrated with other health interventions and national immunization programs expand to include more vaccines and target a broader range of age and population groups, it will be imperative that essential planning and monitoring practices at district and health center levels - which underpin strong RI systems - are strengthened, to enhance protection of all children against vaccine-preventable diseases.

## Conflict of interest

All authors have no conflicts of interest to declare.
